# Effect of total ligamentum flavum removal on increased motion and reduced stiffness: an *ex vivo* biomechanical study in a lamb spine model

**DOI:** 10.3389/fmed.2026.1772268

**Published:** 2026-06-10

**Authors:** Bilal Aykaç, Mustafa Dinç, Recep Karasu, Hünkar Çağdaş Bayrak, Kadri Yıldız

**Affiliations:** 1 Department of Orthopedics and Traumatology, Bursa City Hospital, Bursa, Türkiye; 2 Department of Orthopedics and Traumatology, Bursa Yüksek İhtisas Training and Research Hospital, Bursa, Türkiye; 3 Atakent Private Surgical Medical Center, Bursa, Türkiye

**Keywords:** flavectomy, laminotomy, ligamentum flavum, lumbar spine biomechanics, segmental stability

## Abstract

**Background:**

The ligamentum flavum (LF) is a key component of the posterior tension band and contributes critically to lumbar segmental stability. Although laminotomy and flavectomy are widely performed during lumbar decompression, their isolated biomechanical effects remain incompletely characterized, particularly in controlled ex vivo models with validated similarity to human lumbar mechanics and procedure-specific experimental frameworks. This study investigated whether complete LF removal increases motion and decreases stiffness beyond the effects of laminotomy alone.

**Methods:**

Twenty-four lamb L4–L5 functional spinal units were randomized into three groups: Control (*n* = 8), Laminotomy (*n* = 8), and Laminotomy + Total Flavectomy (*n* = 8). Standardized unilateral laminotomy and bilateral flavectomy were performed. Non-destructive mechanical testing was conducted under axial compression (400 N) and pure-moment loading (8.4 N·m) in lateral bending, flexion, and extension. Displacement and stiffness values were derived from force–displacement and moment–angle curves. Group differences were assessed using Kruskal–Wallis and Dunn–Bonferroni tests, with η^2^ effect sizes reported.

**Results:**

Total flavectomy produced the greatest biomechanical alterations across all loading directions. Compared with controls, the Laminotomy + Flavectomy group demonstrated significantly increased displacement in axial compression (*p* = 0.001), left bending (*p* = 0.001), flexion (*p* < 0.001), and extension (*p* = 0.003). Stiffness was significantly reduced in left bending (*p* < 0.001), flexion (*p* = 0.004), and extension (*p* = 0.016). Isolated laminotomy caused only minor, non-significant changes in most planes. Effect sizes ranged from moderate to large (η^2^ = 0.24–0.71), indicating biomechanically meaningful destabilization.

**Conclusion:**

Total removal of the LF significantly increases motion and decreases stiffness under multiple loading conditions, highlighting its essential role in posterior tension band stability. While laminotomy alone yields minimal biomechanical impact, combining it with flavectomy substantially compromises segmental stability. LF-preserving strategies should be prioritized whenever feasible to reduce the risk of postoperative instability.

## Introduction

The ligamentum flavum (LF) plays a central role in maintaining segmental stability and regulating physiological spinal motion through its unique composition of elastin-rich fibers. This structural specialization allows the LF to restore interlaminar tension during flexion, prevent buckling during extension, and function synergistically with the posterior ligamentous complex to limit excessive vertebral translation (1, 2). Degeneration, hypertrophy, or surgical removal of the LF alters this tension–stability balance, potentially increasing segmental range of motion, facet loading, and shear forces—key contributors to postoperative instability (3).

Surgical decompression procedures targeting lumbar stenosis or disc herniation frequently involve partial removal of posterior elements. While laminotomy preserves stabilizing structures, complete flavectomy necessitates wider exposure and removal of the entire LF, which may compromise biomechanical integrity. Prior *in vitro* and cadaveric studies have demonstrated that excision of posterior ligamentous structures, including the LF, increases flexion–extension mobility and diminishes stiffness, but available data remain inconsistent and highly dependent on the exact surgical technique, vertebral level, and animal model used (4–6).

Animal spine models—particularly lamb and sheep lumbar spines—have been widely adopted due to their anatomical and biomechanical similarity to humans, with validated use in studies involving discectomy, facetectomy, laminectomy, and interbody fusion (7, 8). However, despite well-established use of these models, the isolated biomechanical effect of total LF removal compared with laminotomy alone has not been directly quantified in any lamb spine model. Existing studies either combine flavectomy with additional posterior decompression procedures or focus solely on disc- or facet-based interventions, making it difficult to attribute biomechanical changes specifically to LF excision.

Furthermore, although laminotomy is widely considered a motion-preserving approach, it constitutes the initial surgical corridor for flavectomy. Whether the bony defect created by laminotomy itself alters spinal biomechanics—and how this compares with the more radical total flavectomy—remains poorly understood. A clear understanding of how these two commonly performed procedures independently influence spinal motion and stiffness is essential for minimizing postoperative instability, especially in minimally invasive spine surgery where posterior tension band preservation is a major consideration (9).

Therefore, an important methodological gap persists regarding the quantifiable biomechanical consequences of laminotomy versus total flavectomy, particularly under controlled loading conditions in a validated animal model. Although prior cadaveric and finite element studies have examined posterior element resection in broader contexts, few have directly isolated and compared the independent biomechanical contributions of laminotomy and ligamentum flavum removal within a standardized experimental framework (4–6, 10, 11).

In this context, the present study provides a controlled, procedure-specific comparison by evaluating laminotomy alone and laminotomy combined with total flavectomy under identical loading conditions in an ovine lumbar spine model. The incremental value of this work lies not in redefining established biomechanical principles, but in providing refined, quantitative, and stepwise validation of the distinct contribution of ligamentum flavum resection to segmental motion and stiffness.

The objective of this experimental study was to evaluate the effects of laminotomy and total flavectomy on displacement and stiffness in a lamb lumbar spine model under compression lateral bending, flexion, and extension loading. We hypothesized that: laminotomy would produce minimal changes in segmental biomechanics compared to intact controls, whereas total flavectomy would significantly increase segmental motion and decrease stiffness due to disruption of the posterior ligamentous complex. By isolating these procedures under standardized mechanical testing, this study aims to clarify their differential impact on spinal stability and provide biomechanical evidence to guide surgical decision-making.

## Materials and methods

### Ethical approval

No live animals were used in this ex vivo biomechanical study. The Bursa Uludağ University Animal Experiments Local Ethics Committee (HADYEK) reviewed the study and, in its official correspondence (Reference No: B.30.2.ULU.0.8Z.00.00, dated 19.11.2025), confirmed that ethical approval was not required because the work involved only the use of lamb spine tissues obtained post-mortem from a licensed abattoir.

This determination was made in accordance with Article 8(k) of the national Regulation on the Working Procedures and Principles of Animal Experiments Ethics Committees (Official Gazette No. 28914, 15 February 2014), which exempts studies using tissues obtained after routine slaughter from ethical approval requirements.

All procedures complied with institutional, national, and international guidelines governing the ethical use of biological materials, including EU Directive 2010/63/EU, ICMJE recommendations, and ARRIVE 2.0 guidelines (10–12).

### Study design

This controlled laboratory experiment was designed to investigate the biomechanical effects of laminotomy and total flavectomy on lumbar spinal stability. The study followed internationally recognized standards for spine biomechanics research described by Wilke et al. (13, 14), emphasizing rigorous control of specimen hydration, alignment, preconditioning, and reproducibility. The surgical technique served as the independent variable, while displacement and stiffness across multiple loading directions were defined as dependent variables.

### Specimen procurement and preparation

Fresh lumbar spines were harvested from skeletally mature lambs (8–10 months old) immediately post-slaughter at a licensed abattoir following veterinary inspection. Spinal columns were vacuum-sealed and stored at −20 °C. Freezing at this temperature has been shown to preserve structural and mechanical properties of spinal ligaments and discs (15), and more recent studies confirm that low-temperature storage maintains the mechanical integrity of elastin-rich tissues such as the ligamentum flavum and interspinous ligaments (16–18).

Before testing, the specimens were thawed for 12 h at 24 °C. Continuous hydration was maintained throughout preparation and testing using saline-soaked gauze, as dehydration is known to significantly impair viscoelastic and stiffness characteristics of ligaments (14, 19, 20). Soft tissues were removed while preserving the ligamentum flavum, facet capsules, interspinous and supraspinous ligaments, and intervertebral discs. Functional spinal units (FSUs) were standardized by isolating the L4–L5 motion segment, and PMMA embedding of L4 superiorly and L5 inferiorly was performed in neutral anatomical alignment. Neutral alignment was defined as the orientation in which the FSU exhibited no rotation or translation under zero-load, consistent with established biomechanical norms (21, 22).

### Group allocation

Twenty-four (n:24) L4–L5 functional spinal units were randomly assigned into three experimental groups with eight (n:8) specimens each: an intact control group, a laminotomy group (Group 1) in which a unilateral left-sided laminotomy was created without ligamentum flavum removal, and a laminotomy plus total flavectomy group (Group 2) in which laminotomy was followed by bilateral ligamentum flavum excision. A computer-generated randomization sequence was used, and all specimens were coded by an independent researcher to ensure blinding during biomechanical testing.

### Surgical procedures

All procedures were performed by an experienced spine surgeon. To ensure standardization, the surgeon completed four pilot procedures on non-study FSUs prior to the experiment, consistent with surgical reproducibility practices described in experimental spine research (14, 23, 24). Laminotomy boundaries were defined using consistent anatomical landmarks (inferior L4 lamina, superior L5 lamina, medial facet border). After each procedure, resection extent was visually verified by an independent observer, ensuring uniformity across specimens.

**Figure 1:**
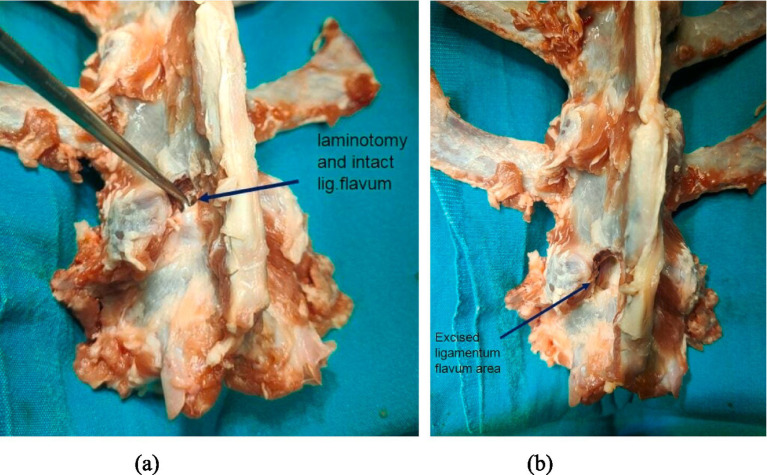
Surgical preparation of the lumbar motion segments prior to biomechanical testing. **(a)** The laminotomy-only specimen showing a preserved ligamentum flavum (arrow) following unilateral laminotomy. These images verify the standardized surgical differentiation between study groups used in all biomechanical analyses. **(b)** The laminotomy + total flavectomy specimen demonstrating complete excision of the ligamentum flavum (arrow), confirming full removal of the posterior ligamentous structure.

The laminotomy was created using a high-speed drill and Kerrison rongeur, forming a standard minimally invasive corridor while preserving the facet joints, pars interarticularis, supraspinous and interspinous ligaments, following techniques described for MIS lumbar decompression (25–27). For total flavectomy, the ligamentum flavum was dissected and excised en bloc. The contralateral ligamentum flavum was removed via a sublaminar approach without further bone resection, consistent with unilateral-approach bilateral decompression methods (28–31) (see Figure 1).

### Biomechanical testing

Biomechanical measurements were performed using a Shimadzu AG-X Plus universal testing machine equipped with a 250-kN load cell, which has been validated in prior lumbar spine mechanical investigations (3, 14, 32). The laboratory was maintained at 24 °C, and specimens remained continuously hydrated throughout testing.

All FSUs underwent a preconditioning protocol of 10 sinusoidal cycles between 5 and 50 N at 0.1 Hz to eliminate viscoelastic creep, following classical recommendations by Panjabi et al. (33). Testing was conducted in the fixed sequence of axial compression, right and left lateral bending, and finally flexion/extension, a sequence shown to minimize cumulative tissue damage (21, 34, 35).

**Figure 2:**
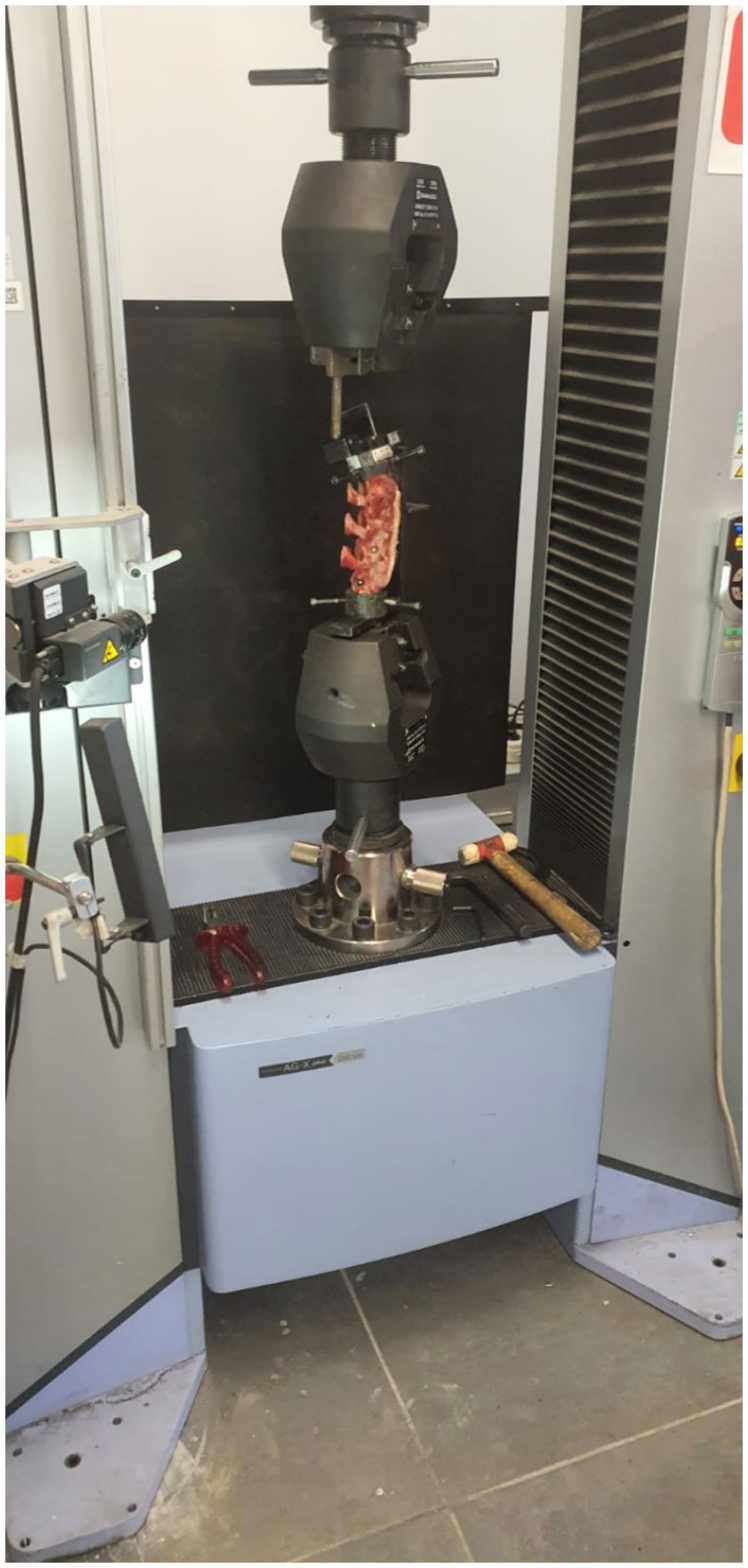
Experimental biomechanical testing setup used for non-destructive axial compression and pure-moment loading of L4–L5 functional spinal units. The specimen was mounted in a Shimadzu AG-X Plus universal testing machine using customized upper and lower fixtures, ensuring rigid fixation and alignment of the motion segment. Axial compression loads (400 N) and pure-moment loads (8.4 N·m) in flexion, extension, and lateral bending were applied under displacement control. High-precision optical markers were attached to the vertebral bodies for continuous tracking of displacement and angular deformation during testing. The standardized setup ensured consistent load distribution and minimized off-axis loading across all specimens.

Axial compression was applied at 20 N/s until 400 N was reached, with displacement continuously recorded. Compression stiffness was calculated from the linear region of the force–displacement curve (typically 100–300 N), using R^2^ ≥ 0.98 to ensure linearity. Lateral bending was assessed using pure-moment loading to generate 8.4 N·m by applying force 60 mm from the central axis, following pure-moment protocols validated in classic biomechanical literature (4, 34, 36, 37). Flexion and extension were assessed at the same 8.4 N·m magnitude, with angular displacement measured using a high-sensitivity digital extensometer. Stiffness in bending and flexion/extension was extracted from the linear portion of the moment–angle curves (0–6 N·m), consistent with established biomechanical extraction methods (36, 37) (Figure 2). The biomechanical engineer performing all tests was blinded to group allocation. Coding and PMMA embedding masked procedural differences, a blinding method frequently implemented in ex vivo spine biomechanics studies (14, 38).

### Data acquisition

All force, displacement, and moment data were captured digitally using Trapezium-X software. Displacement was defined as the maximum translation or rotation under applied load or moment, while stiffness represented the slope of the linear elastic region of each mechanical curve. The definitions and extraction methods were standardized across all specimens to ensure reproducibility.

### Statistical analysis

Statistical analyses were conducted using IBM SPSS Statistics version 27.0 (IBM Corp., Armonk, NY, USA). The distributional characteristics of the dataset were assessed using the Shapiro–Wilk test, which is recommended for small- to moderate-sized samples due to its superior sensitivity in detecting deviations from normality. Examination of the results revealed that several variables did not meet normal distribution assumptions. Considering the modest sample size and the non-normality of the data, all group comparisons were performed using the nonparametric Kruskal–Wallis test, which is appropriate for comparing multiple independent groups when normality and homogeneity assumptions are not satisfied. When significant group differences were identified, *post-hoc* pairwise analyses were conducted using the Dunn–Bonferroni correction to control for Type I error in multiple comparisons. Statistical significance was set at *p* < 0.05.

The sample size for this study was determined *a priori* using the Resource Equation Method, a widely accepted approach in animal experimentation and biomechanical research when large sample sizes are impractical or when limited biological material is available (39, 40). According to this method, the use of eight specimens per group in a three-group design falls within the recommended E-value range (10–20), confirming that the study design provides an appropriate balance between statistical power and ethical use of specimens. Moreover, the sample size of eight specimens per group is consistent with previously published ex vivo lumbar spine biomechanics studies using similar animal models (14, 41), and the large effect sizes observed (η^2^ = 0.24–0.71) support that the study was adequately powered to detect biomechanically meaningful differences.

## Results

A total of 24 lumbar motion segments were analyzed across three groups: control (*n* = 8), laminotomy (*n* = 8), and laminotomy + flavectomy (*n* = 8). Kruskal–Wallis testing identified significant overall group differences across multiple displacement and stiffness parameters (*p* < 0.05); however, *post hoc* pairwise comparisons revealed that statistically significant differences between specific groups were not uniform across all parameters. Detailed numerical values are provided in Table 1.

**Table 1:** Biomechanical displacement and stiffness values across study groups under axial compression, lateral bending, flexion, and extension loading.

Measurement	Control	Laminotomy	Flavectomy	*p*-value	Effect Size (η^2^)
Displacement					
Compression	0.59 (0.54–0.92) [0.64]	0.84 (0.51–1.17) [0.84]	1.09 (0.66–1.55) [1.11]	0.002	0.495
Right Bending	7.77 (4.99–12.80) [8.32]	13.27 (8.63–17.65) [13.21]	12.36 (7.57–25.97) [14.84]	0.011	0.338
Left Bending	14.77 (7.76–19.08) [13.83]	16.14 (13.07–20.28) [16.11]	20.63 (18.28–24.43) [20.92]	<0.001	0.583
Flexion	10.45 (9.32–14.84) [11.30]	13.73 (10.10–14.90) [13.23]	19.93 (14.68–22.37) [19.34]	<0.001	0.668
Extension	5.09 (3.40–6.17) [5.11]	5.53 (3.48–7.28) [5.70]	11.94 (5.10–14.83) [10.92]	0.004	0.420
Stiffness					
Compression	640.13 (457.06–826.56) [641.22]	471.56 (377.96–716.32) [499.21]	483.98 (346.01–740.34) [497.35]	0.043	0.238
Right Bending	1.63 (1.31–2.20) [1.70]	1.06 (0.63–1.16) [0.97]	0.95 (0.74–1.08) [0.92]	<0.001	0.642
Left Bending	1.34 (1.02–1.68) [1.34]	1.08 (0.92–1.19) [1.05]	0.75 (0.54–0.99) [0.74]	<0.001	0.713
Flexion	0.80 (0.63–1.01) [0.79]	0.66 (0.37–0.80) [0.63]	0.52 (0.35–0.81) [0.52]	0.006	0.460
Extension	1.69 (1.50–2.42) [1.84]	1.65 (1.53–2.26) [1.77]	1.02 (0.79–1.73) [1.14]	0.014	0.343

Values are presented as median (min–max) [mean]. Statistical comparisons were performed using the Kruskal–Wallis test due to non-normal data distribution, followed by Dunn–Bonferroni post-hoc analysis for pairwise group comparisons. Effect sizes were computed using eta-squared (η^2^), where values of 0.01–0.06 represent small, 0.06–0.14 moderate, and >0.14 large effects, indicating the magnitude of biomechanical differences among groups. Significant *p*-values (<0.05) indicate measurable alterations in spinal motion segment behavior following laminotomy and total flavectomy. While overall group differences were identified using the Kruskal–Wallis test, statistical significance between specific groups was determined based on Dunn–Bonferroni *post hoc* comparisons.

## Specific findings by loading mode

### Axial compression

**Figure 3:**
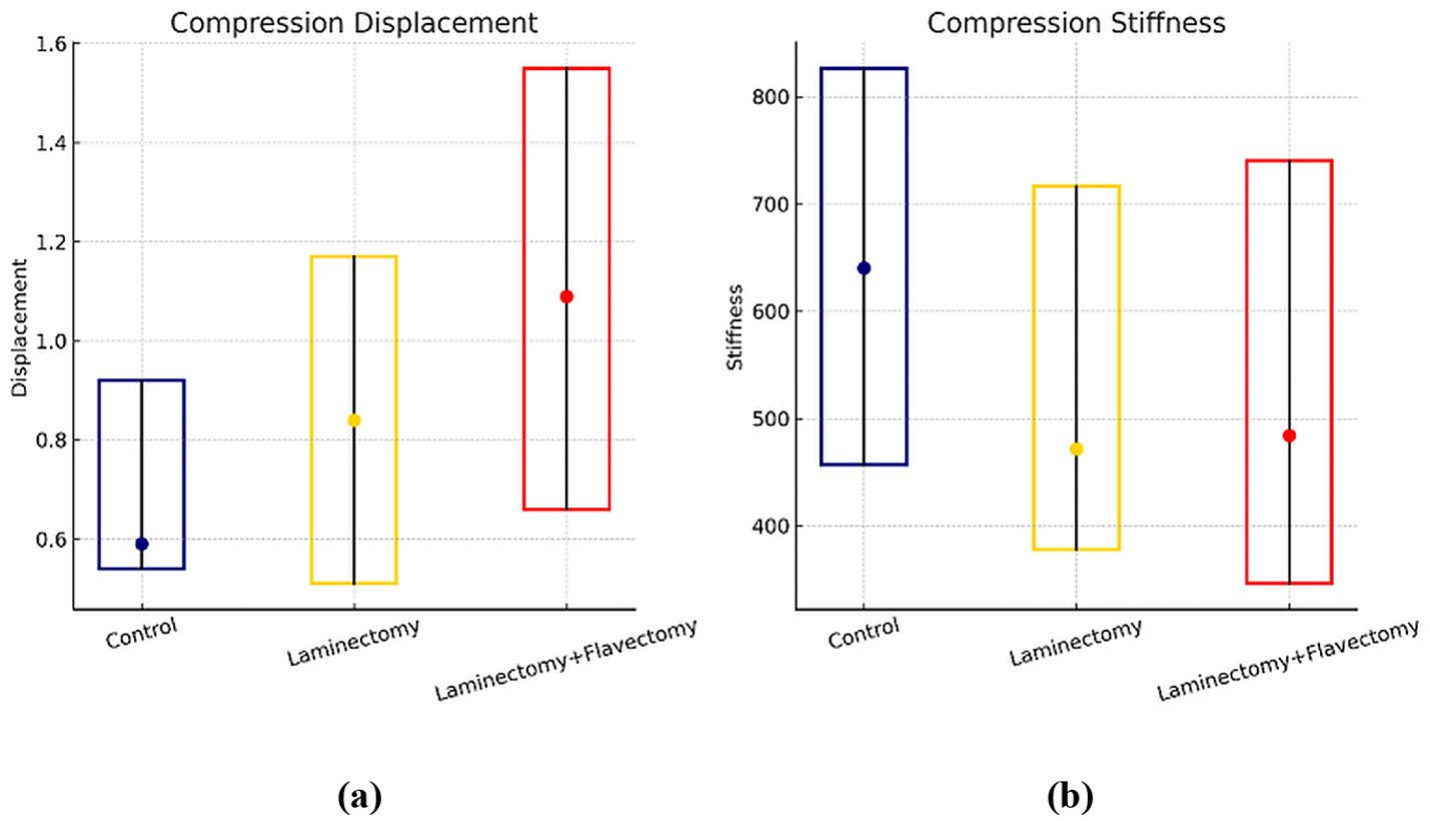
Biomechanical response of lumbar motion segments under axial compression. **(a)** Compression displacement (mm) and **(b)** compression stiffness (N/mm) for the control, laminotomy, and laminotomy + total flavectomy groups. Data are presented as median values with interquartile ranges, with group means illustrated by colored markers. Statistical comparisons were performed using the Kruskal–Wallis test followed by Dunn–Bonferroni *post hoc* analysis.

Compression displacement showed a significant overall group effect (*p* = 0.002, η^2^ = 0.495), with post hoc analysis identifying a significant difference exclusively between the Control and Laminotomy + Flavectomy groups (Adj. *p* = 0.001); neither the Control vs. Laminotomy nor the Laminotomy vs. Laminotomy + Flavectomy comparisons reached significance. Although an overall group difference was also detected for compression stiffness (*p* = 0.043, η^2^ = 0.238), no pairwise comparison achieved significance after Bonferroni correction. Results are illustrated in Figure 3.

### Lateral bending (right and left)

For right lateral bending, both surgical groups demonstrated significantly greater displacement and lower stiffness than the Control group (all Adj. *p* < 0.05), with no significant difference between the two surgical groups for either parameter (Adj. *p* = 1.000 for both). Notably, this pattern — in which isolated laminotomy demonstrated biomechanical changes similar to those observed after laminotomy + flavectomy — was specific to the right (ipsilateral) side, possibly reflecting asymmetric load transfer related to the unilateral laminotomy approach.

**Figure 4:**
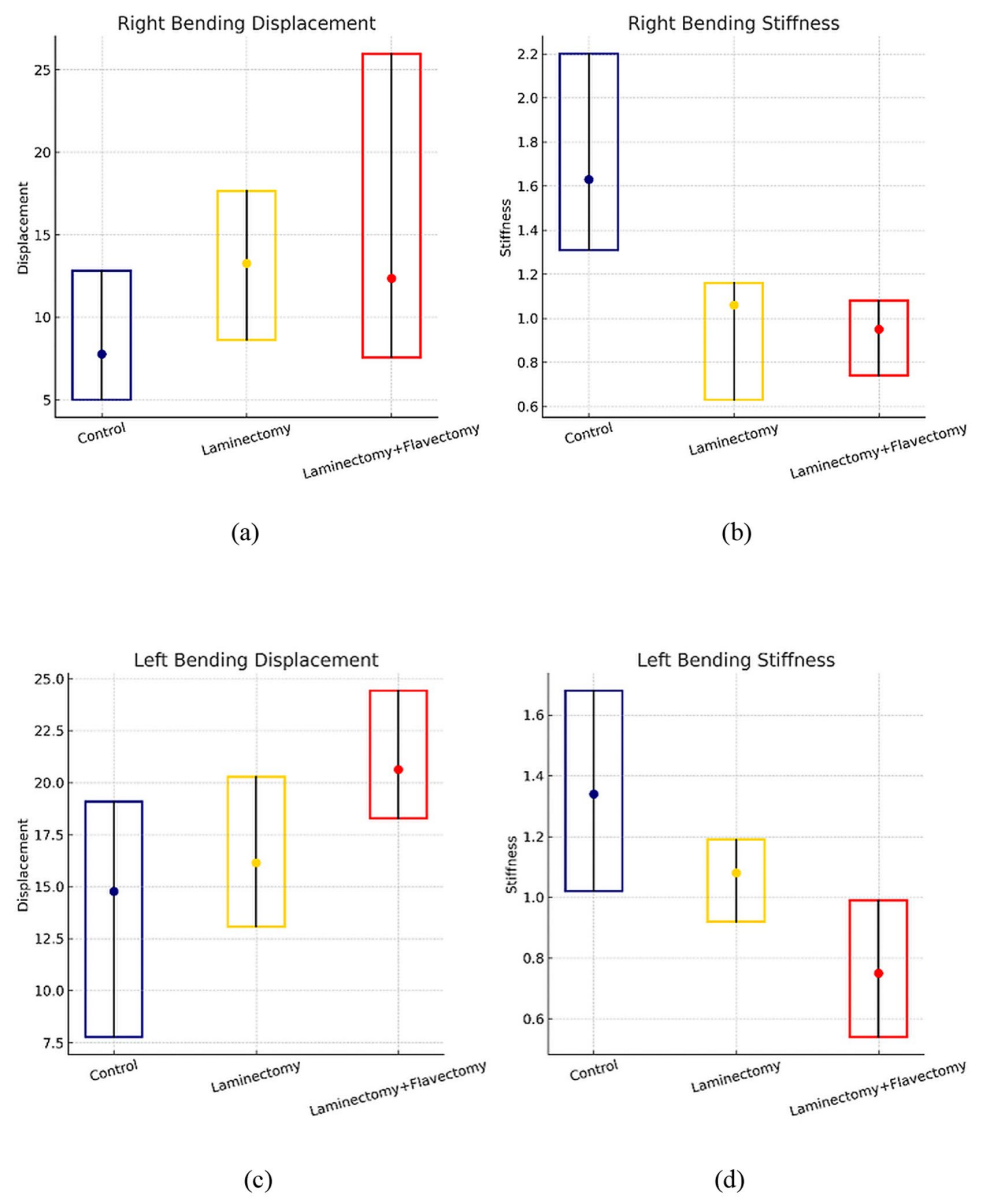
Segmental response to right and left lateral bending. **(a)** Right lateral bending displacement (mm), **(b)** right lateral bending stiffness (N/mm), **(c)** left lateral bending displacement (mm), and **(d)** left lateral bending stiffness (N/mm) for the control, laminotomy, and laminotomy + total flavectomy groups. Data are presented as median values with interquartile ranges, with group means indicated by colored markers. Statistical comparisons were performed using the Kruskal–Wallis test followed by Dunn–Bonferroni *post hoc* analysis.

For left lateral bending, the Laminotomy + Flavectomy group exhibited significantly greater displacement and lower stiffness than both the Control and Laminotomy groups (all Adj. *p* ≤ 0.009), whereas the Laminotomy group did not differ significantly from the Control for either parameter. These findings are illustrated in Figure 4.

### Flexion and extension

**Figure 5:**
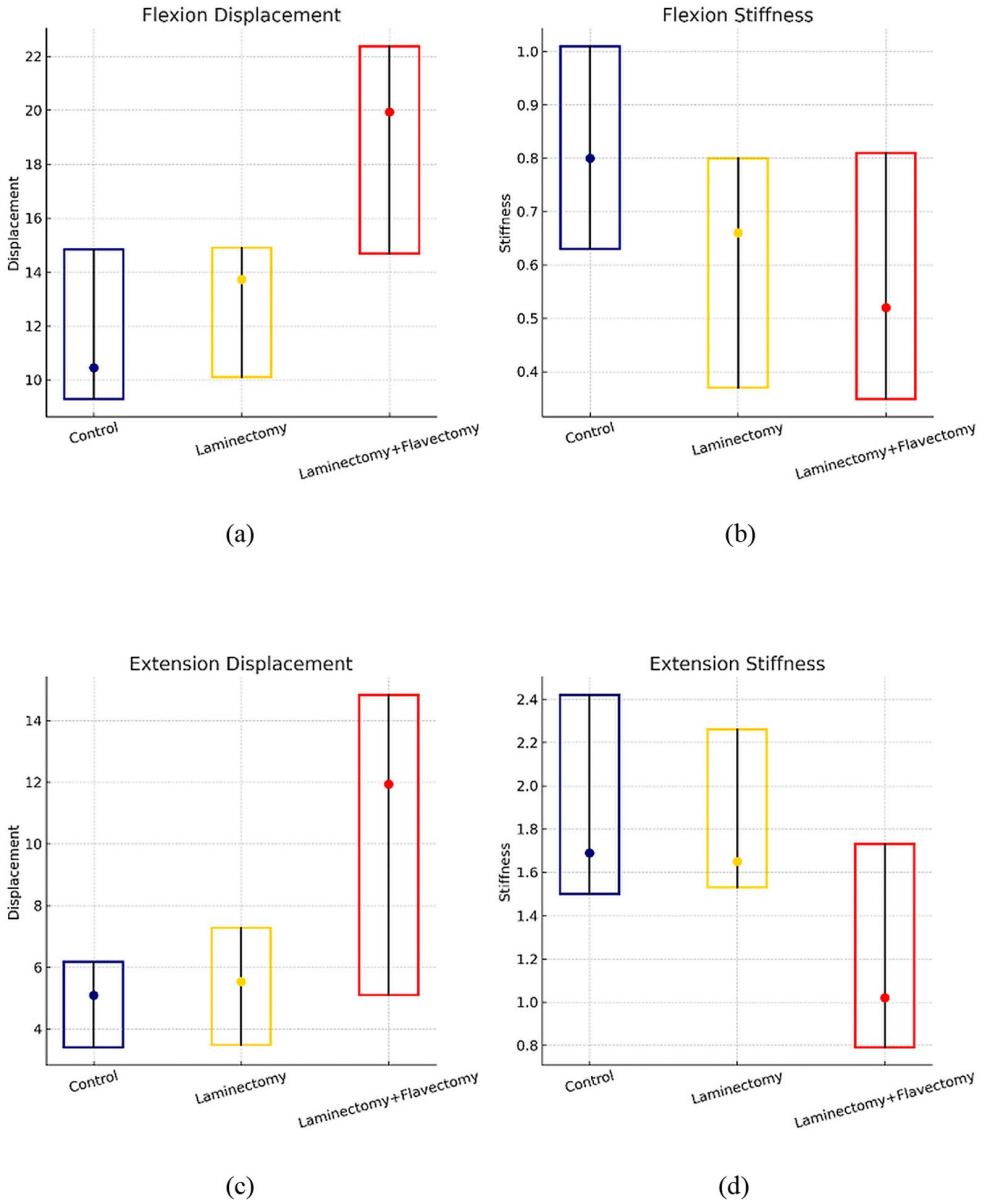
Segmental response to flexion and extension. **(a)** Flexion displacement (mm), **(b)** flexion stiffness (N/mm), **(c)** extension displacement (mm), and **(d)** extension stiffness (N/mm) for the control, laminotomy, and laminotomy + total flavectomy groups. Data are presented as median values with interquartile ranges, with group means indicated by colored markers. Statistical comparisons were performed using the Kruskal–Wallis test followed by Dunn–Bonferroni *post hoc* analysis.

For flexion, the Laminotomy + Flavectomy group demonstrated significantly greater displacement than both the Control (Adj. *p* < 0.001) and Laminotomy (Adj. *p* = 0.024) groups, and significantly lower stiffness than the Control (Adj. *p* = 0.004); no other pairwise comparisons reached significance. For extension, significant differences were confined to the Control vs. Laminotomy + Flavectomy comparison for both displacement (Adj. *p* = 0.003) and stiffness (Adj. *p* = 0.016). In both loading modes, isolated laminotomy did not produce statistically significant biomechanical alterations relative to controls. These findings are illustrated in Figure 5.

## Discussion

### Summary of main findings

This controlled *ex vivo* biomechanical study demonstrated that both laminotomy and, more prominently, total flavectomy significantly altered lumbar motion segment mechanics. Across all loading modes — including axial compression, lateral bending, flexion, and extension — segmental displacement increased and stiffness decreased compared with the control group. These changes were most pronounced in the laminotomy + flavectomy group, indicating that removal of the ligamentum flavum (LF) may substantially compromise posterior tension band integrity. Effect size analyses (η^2^ = 0.24–0.71) further confirmed that the observed differences were biomechanically substantial and clinically meaningful, reflecting a measurable loss of segmental stability. However, not all statistically significant overall group effects were supported by significant pairwise differences between specific groups, and these findings should therefore be interpreted with caution when considering direct comparisons between individual groups.

### Biomechanical role of the ligamentum flavum

The LF is a key stabilizing structure of the lumbar spine, contributing to elastic recoil, resistance to buckling, and maintenance of interlaminar tension during physiological movement. Our findings are consistent with those of Anderson et al. (42), who reported that complete flavectomy increases segmental range of motion and predisposes motion segments to instability through disruption of the posterior ligamentous complex. Similarly, Pizones et al. (43) showed that LF rupture or resection frequently occurs alongside interspinous ligament injury, exacerbating flexion instability and pathological translation. In our study, the greatest increases in motion — especially during flexion and lateral bending — occurred following flavectomy, supporting the notion that the LF plays a crucial role in resisting excessive posterior-column deformation.

### Comparison with previous biomechanical and surgical studies

Our findings align with prior cadaveric and *in vitro* studies emphasizing the biomechanical importance of preserving posterior osseoligamentous structures. Karakaşlı et al. (41) demonstrated that maintaining posterior bony and ligamentous integrity during minimally invasive lumbar procedures reduces the risk of postoperative instability, facet degeneration, and disc space narrowing. Similarly, Kettler et al. (44) showed that decompressive interventions such as fenestration and discectomy increase lateral bending and flexion mobility while reducing intradiscal pressure — alterations that may shift load transfer and promote degenerative progression. The present study extends this body of evidence by clearly distinguishing the independent biomechanical effects of isolated laminotomy and total flavectomy: while laminotomy alone induced only modest changes, combining it with complete LF excision markedly amplified segmental destabilization.

Recent clinical evidence further complements these biomechanical findings. In a retrospective cohort study of 299 patients undergoing L4–L5 posterolateral arthrodesis for degenerative spondylolisthesis and stenosis, Aimar et al. (45) compared conventional decompression with an over-level flavectomy (OLF) technique including prophylactic L3–L4 flavectomy. The OLF group exhibited significantly lower rates of cranial adjacent segment disease (CASD) and better clinical outcomes at five-year follow-up, without increasing iatrogenic instability or accelerating adjacent-level disc degeneration. These findings are relevant to our results in two respects: first, they suggest that the LF’s role is not uniformly destabilizing — its resection can be performed safely in carefully selected contexts where the goal is to prevent future stenosis rather than preserve motion; second, the absence of increased instability in the OLF group aligns with our observation that laminotomy alone produced minimal biomechanical alterations, whereas complete flavectomy at the index level resulted in significant destabilization. Taken together, these data support a nuanced, indication-specific approach to LF resection.

Beyond confirming previously reported biomechanical trends, the present study provides a more refined and procedure-specific evaluation of posterior element resection. While prior studies have often assessed combined decompression techniques or heterogeneous surgical modifications, our design enables direct comparison of laminotomy and total flavectomy within the same standardized model and loading conditions (41, 46, 47). allowing clearer attribution of biomechanical changes specifically to LF removal and minimizing confounding related to multi-structure resection.

Several contemporary biomechanical studies further support our findings. Recent finite element analyses have shown that wide laminectomy significantly increases flexion–extension range of motion and reduces stiffness, confirming that posterior element removal alters segmental stability even without isolating the LF (46). A systematic review of lumbar decompression biomechanics similarly reported that posterior element resection consistently increases motion, reduces load-bearing capacity, and may predispose the motion segment to accelerated degeneration (47). Additionally, tensile testing of the human LF has demonstrated substantial resistance to shear and anterior translation, with age- and degeneration-related changes further influencing segmental behavior — supporting our observation that LF removal disrupts normal flexion mechanics (48). These findings are consistent with classic work showing that posterior ligament resection disproportionately increases the neutral zone compared with facet joint removal (6, 49) and that posterior soft tissues contribute more to low-load lumbar stability than bony elements (6, 50). Collectively, this body of evidence reinforces the stabilizing role of the LF and supports the validity of the lamb spine model used in the present study (8, 51–53). These biomechanical alterations may not only pose a risk of immediate instability but could also predispose the segment to long-term degenerative changes through altered stress distribution across the facet joints and intervertebral disc.

### Mechanistic interpretation of biomechanical changes

The observed biomechanical alterations can be explained by disruption of the posterior tension band complex. The LF contributes an estimated 40–60% of the resistance to flexion due to its high elastin content and elastic recoil properties (50, 54). ts removal eliminates a major passive stabilizer responsible for limiting interlaminar distraction and maintaining the neutral zone. According to Panjabi’s (55) subfailure injury cascade, resection of posterior ligaments increases segmental laxity, alters the instantaneous axis of rotation, and produces abnormal coupled motion patterns — all of which align with the increased flexion and lateral bending displacement observed in our study. Biomechanical evidence further confirms that posterior ligament disruption reduces stiffness under low-load conditions and expands the neutral zone (56, 57), with posterior element removal disproportionately reducing stiffness in the neutral zone compared with the elastic zone (58, 59) — consistent with the pronounced stiffness reductions observed after flavectomy in our dataset. Because the LF also prevents excessive buckling during extension, its removal explains the extension stiffness deficits observed in the present study.

A notable asymmetry was observed between right and left lateral bending responses. On the right (ipsilateral) side, both surgical groups demonstrated comparable increases in displacement and reductions in stiffness relative to controls, with no significant difference between them. This pattern suggests that the ipsilateral bony defect created by unilateral laminotomy constitutes the dominant source of mechanical disruption during ipsilateral bending, potentially masking the incremental contribution of LF removal. In contrast, left (contralateral) lateral bending revealed a clear stepwise pattern, with significant differences emerging only in the Laminotomy + Flavectomy condition. Since the contralateral posterior cortex remained structurally intact, the stabilizing contribution of the LF became biomechanically distinguishable on this side, allowing its removal to produce a measurable and independent destabilizing effect. This directional asymmetry underscores the importance of reporting ipsilateral and contralateral loading responses separately in unilateral decompression models.

Thus, the combined effects of bony removal (laminotomy) and ligamentous removal (flavectomy) create a segment that is biomechanically vulnerable to increased translation, shear forces, and abnormal motion pathways, potentially predisposing the spinal unit to instability.

### Neutral zone expansion as a mechanism of instability

The observed biomechanical alterations can be further understood through Panjabi’s neutral zone (NZ) concept. The NZ is the region of intervertebral motion near the neutral position where minimal ligamentous resistance occurs, and its enlargement is considered a more sensitive indicator of spinal instability than an isolated increase in range of motion. In the intact spine, the LF contributes substantially to maintaining a narrow NZ by providing elastic recoil and resisting interlaminar distraction under low loads. Following total flavectomy, loss of this passive tension leads to NZ expansion, meaning that larger angular displacements occur before the spine engages its stiffer elastic zone. Our finding that flexion displacement increased by approximately 90% (median: Control 10.45 mm vs. Laminotomy + Flavectomy 19.93 mm) while flexion stiffness decreased by approximately 35% is consistent with NZ enlargement. Notably, the laminotomy-only group showed no statistically significant increase in flexion displacement or stiffness reduction compared with controls, suggesting that the LF — rather than the bony laminotomy defect — is the primary restraint within the NZ during flexion and extension. This interpretation directly links our results to established spine stability theory and reinforces the importance of LF preservation for maintaining the neutral zone.

### Clinical interpretation and surgical implications

Clinically, these findings suggest the potential importance of minimizing unnecessary resection of ligamentous and bony components during lumbar decompression procedures, including endoscopic spine surgery (ESS) and lumbar microdiscectomy (LMD). While laminotomy alone produced moderate biomechanical changes, the addition of total flavectomy resulted in significant increases in displacement and marked reductions in stiffness across all motion planes.

These data underscore the essential stabilizing function of the LF and suggest that complete excision—particularly in the absence of severe hypertrophy or adhesions—may predispose patients to postoperative instability, recurrent pain, or accelerated degenerative progression. However, these findings should be interpreted as reflecting biomechanical trends observed under controlled experimental conditions, rather than as direct predictors of postoperative instability in human patients. Differences in spinal anatomy, posture, and physiological loading between ovine and human models must be considered when translating these results to clinical practice. Therefore, surgical strategies such as under-the-facet flavum preservation or partial flavectomy in cases without severe hypertrophy may help maintain postoperative biomechanical balance while achieving adequate decompression.

In addition, patient-specific factors such as age, segmental morphology, and the degree of degenerative changes may further influence the decision to preserve or resect the ligamentum flavum, as these variables can modify baseline segmental stiffness and biomechanical response to decompression.

From a surgical guidance perspective, the following strategies are recommended based on the present biomechanical findings: (1) In patients without severe LF hypertrophy, calcification, or epidural adhesions, complete flavectomy may not be necessary and should be avoided unless required for adequate decompression; partial flavectomy or under-the-facet flavum preservation should be considered, as these may provide sufficient neural decompression while maintaining posterior tension band integrity. (2) When bilateral decompression is indicated, unilateral laminotomy with contralateral sublaminar flavectomy — as performed in our model — is biomechanically preferable to bilateral flavectomy with wider bone resection, as the latter may exacerbate segmental instability. (3) In younger patients or those with pre-existing segmental instability (e.g., degenerative spondylolisthesis), preserving the LF is particularly critical to reducing the risk of postoperative instability and adjacent segment degeneration.

## Strengths and limitations

This study has several strengths, including its controlled *ex vivo* experimental design, standardized mechanical testing protocol, and effect-size–based analysis, which allowed quantification of the magnitude of biomechanical alterations. The isolated comparison of laminotomy and total flavectomy provided a clear understanding of each procedure’s independent effects on spinal stability.

However, limitations must also be acknowledged. Although lamb lumbar spines provide validated anatomical and biomechanical similarity to human specimens, the absence of muscular forces, ligamentous tensioning, and soft tissue constraints in this ex vivo model limits the translation of findings to *in vivo* conditions. Only static loading was evaluated; dynamic or cyclic loading may offer more clinically relevant insights into fatigue behavior and long-term stability. Furthermore, the fixed loading sequence (axial compression → lateral bending → flexion/extension) may have introduced cumulative viscoelastic effects, as sequential testing does not allow full recovery of the viscoelastic tissues between loading modes. Consequently, the observed stiffness reductions could partly reflect time- and history-dependent mechanical behavior rather than surgical disruption alone. Additionally, axial rotational loading was not assessed, and cyclic fatigue loading protocols were not included. These limitations may result in underestimation of three-dimensional instability and limit assessment of time-dependent mechanical behavior under repetitive physiological loading conditions. The absence of histological analysis is another limitation, as microscopic structural changes could help contextualize biomechanical alterations. In addition, species-specific differences in intervertebral disc geometry, ligamentum flavum structure, and physiological loading conditions may influence biomechanical responses, thereby limiting direct extrapolation of these findings to human spinal biomechanics. Another limitation of this study is that the degree of intervertebral disc degeneration was not specifically assessed or stratified. All specimens were obtained from relatively young, healthy lamb spines, and therefore do not reflect the spectrum of disc degeneration commonly observed in patients with lumbar spinal stenosis. Given that disc degeneration can significantly influence segmental biomechanics and contribute to instability, the absence of degenerative disc conditions in this model may limit the direct clinical applicability of the findings. Future studies incorporating varying degrees of disc degeneration would provide a more comprehensive understanding of the combined effects of posterior element resection and disc pathology on spinal stability.

In addition, beyond disc-related factors, age-related and morphology-dependent degenerative changes were not represented in this model. Since these factors may influence both baseline spinal biomechanics and the mechanical consequences of ligamentum flavum resection, their absence may limit the direct clinical applicability of the findings to heterogeneous clinical populations.

Another important limitation is that the potential biomechanical effects of concomitant facet joint resection were not assessed in this study. In clinical practice, lateral recess or foraminal decompression often requires varying degrees of facet remodeling, which may further compromise segmental stability when combined with ligamentum flavum removal. The isolated nature of our surgical model may therefore not fully replicate the complexity of real-world decompressive procedures. Additionally, this study did not account for segment-specific biomechanical differences across the lumbar spine, as findings from L4–L5 may not be directly applicable to other levels (e.g., L1–L2 or L5–S1), which differ in geometry, mobility, and loading characteristics. Furthermore, the sagittal spinal profile (e.g., Roussouly classification) was not considered, despite its potential influence on segmental loading patterns and instability risk. Finally, the model did not incorporate pathological tissue conditions commonly present in surgical candidates—such as ligamentum flavum calcification, marginal osteophyte formation, or advanced facet joint osteoarthritis—which may alter the biomechanical response to posterior element resection and limit direct clinical extrapolation.

## Future directions

Future studies should incorporate dynamic cyclic loading protocols, three-dimensional motion analysis, and human cadaveric models to more accurately approximate physiological conditions. Integrating imaging-based degeneration scoring, histological assessment, and biomechanical behavior would allow more comprehensive characterization of postoperative stability. Additionally, evaluating graded flavectomy (partial vs. complete) may help identify a “safe resection threshold” that balances effective decompression with preservation of biomechanical integrity. Longitudinal studies exploring the cumulative effects of minimally invasive surgical corridors and ligament-sparing techniques would also be valuable. Future studies should incorporate axial rotation testing and cyclic fatigue loading protocols to better simulate physiological spinal mechanics and evaluate long-term stability under repetitive loading conditions. Additionally, future studies should evaluate the combined biomechanical effects of graded facet resection and ligamentum flavum removal, as the extent of facet remodeling required for adequate lateral recess or foraminal decompression may represent an additional and clinically relevant source of segmental instability.

## Conclusion

This study demonstrated that laminotomy and, more prominently, total flavectomy significantly influence lumbar spinal biomechanics by increasing displacement and reducing stiffness across multiple loading directions. These findings support the biomechanical role of the ligamentum flavum and indicate that preservation of posterior ligamentous and bony structures may be beneficial whenever feasible during decompressive spine surgery. Tailoring decompression techniques to achieve neural relief while safeguarding mechanical stability remains a fundamental priority in surgical planning.

However, as muscular contributions, tissue healing responses, and long-term adaptive changes were not accounted for, these findings should be interpreted as suggesting a potential biomechanical risk rather than directly predicting clinical instability.

## Data Availability

The raw data supporting the conclusions of this article will be made available by the authors, without undue reservation.
